# QNetDiff: a quantitative measurement of network rewiring

**DOI:** 10.1186/s12859-024-05702-z

**Published:** 2024-03-18

**Authors:** Shota Nose, Hirotsugu Shiroma, Takuji Yamada, Yushi Uno

**Affiliations:** 1 Graduate School of Engineering, Osaka Prefecture University, Sakai, Japan; 2 Department of Life Science and Technology, Tokyo Institute of Technology, Tokyo, Japan; 3 Graduate School of Informatics, Osaka Metropolitan University, Sakai, Japan; 4 Metagen, Inc., Yamagata, Japan; 5 Metagen Theurapeutics, Inc., Yamagata, Japan; 6 digzyme, Inc., Tokyo, Japan

**Keywords:** Bacterial correlation network, Elimination of false correlations, Feature selection, Metagenomics, Microbiome, Network rewiring

## Abstract

Bacteria in the human body, particularly in the large intestine, are known to be associated with various diseases. To identify disease-associated bacteria (markers), a typical method is to statistically compare the relative abundance of bacteria between healthy subjects and diseased patients. However, since bacteria do not necessarily cause diseases in isolation, it is also important to focus on the interactions and relationships among bacteria when examining their association with diseases. In fact, although there are common approaches to represent and analyze bacterial interaction relationships as networks, there are limited methods to find bacteria associated with diseases through network-driven analysis. In this paper, we focus on rewiring of the bacterial network and propose a new method for quantifying the rewiring. We then apply the proposed method to a group of colorectal cancer patients. We show that it can identify and detect bacteria that cannot be detected by conventional methods such as abundance comparison. Furthermore, the proposed method is implemented as a general-purpose tool and made available to the general public.

## Introduction

Bacteria in the human body have various effects on human health. In particular, identifying the intestinal bacteria associated with human diseases is considered to be effective for elucidating the mechanisms of disease development and developing diagnostic tools for early detection of diseases. For example, the relationship between intestinal bacteria and colorectal cancer is well known [1, 2], and it has been reported that the abundance of bacteria such as *Fusobacterium* and *Solobacterium* increases with the progression of colorectal cancer [3]. In addition, diabetes, atherosclerosis, autism, dementia, Alzheimer’s disease and inflammatory bowel disease have also been linked to intestinal bacteria [4–10].

The most common method of investigating bacteria associated with diseases is to calculate the bacterial composition derived from DNA extracted from stool samples and compare the abundance of each bacteria in the healthy and diseased groups and the changes in abundance. Furthermore, for each of the healthy and diseased groups, a network (*bacterial correlation network*), in which the bacteria are the nodes (vertices) and the correlation coefficients obtained by quantifying the co-occurrence relationship of the bacteria in each group are the links (edges), is constructed. Then the structure of each network is compared, allowing an analysis that takes into account the population structure of the bacteria.

The idea of using bacterial correlation networks to uncover disease features has already been reported [11–14]. For example, there are studies that have confirmed the network changes in the microbiome analysis of diabetes or gastrectomy [4, 15]. However, the information obtained from network dynamics has been used as supplementary information along with comparisons of bacterial abundance. Identifying bacteria using network dynamics is currently very limited.

In general, when the structure of the bacterial communities change, the bacterial correlation network is also considered to be affected. *Rewiring*, where certain bacteria change their co-occurrence relationships with other bacteria, is one of the best-known examples [3, 15, 16]. Bacteria involved in rewiring may be related to the development or progression of the disease. However, quite few methods and tools exist to focus on and quantify such rewiring.

In conducting metagenomic analyses based on the network structure of bacterial communities and the dynamics, one of the significant issues is the noise that arises in constructing bacterial correlation networks. Typically, this noise is caused by an imbalance in the abundances of each one of the bacterial pairs constructing network edges. For such noise, tools such as SparCC [17] are available to reduce it.

On the other hand, in metagenomic analysis, a single DNA read may map to several closely related bacteria with the same score. In this case, standard mapping tools (e.g., bowtie2 and bwa) will either distribute a single read equally to each of all closely related bacteria or randomly map it to one of the closely related bacteria with equal probability. In both cases, the abundance of closely related bacteria will become similar. When the correlation between bacteria is calculated for such data, the correlation of closely related bacteria may be much higher than they actually are, but such *false correlations* cannot be avoided for closely related bacteria. However, there is no practical or effective way has been established for dealing with this noise.

In this paper, we propose a method for extracting characteristic bacteria by quantifying the rewiring between two bacterial correlation networks, to discover and identify bacteria that are likely to be associated with diseases but cannot be detected from changes in abundance alone. At the same time, we propose a new method to reduce and remove noise caused by false correlations, and use this method to preprocess the data. The effectiveness of the proposed method is verified by constructing and applying a *human intestinal bacteria correlation network* based on the metagenomic data from colorectal cancer patients [18]. A Python script that implements a series of processes including above noise removal due to the false correlations and quantification of rewiring is available on GitHub [19].

## Method

This paper proposes a method for presenting candidate bacteria as possible causes of attribute value changes by exploiting structural changes in the bacterial correlation network between two groups with different attribute values. The attribute values are typically a healthy group or a diseased group. When comparing two groups, we focus not only on primary statistics such as the abundance of bacteria or the increase or decrease in the abundance of bacteria, but also on secondary data such as the correlation between bacteria to analyze the network and present candidate bacteria as possible causes of the change in attribute values. To this end, we quantify the network rewiring, in which a particular bacterium changes its co-occurrence relationship with other bacteria.

As a concrete example, we illustrate the method by presenting candidate bacteria that might have strong association with colorectal cancer, when we are given the data of bacterial abundance from the stool samples. Here, we use human colorectal cancer and bacteria as examples, however, we assume that this is a general-purpose method that can be applied to two groups of similar data in general.

**Fig. 1:**
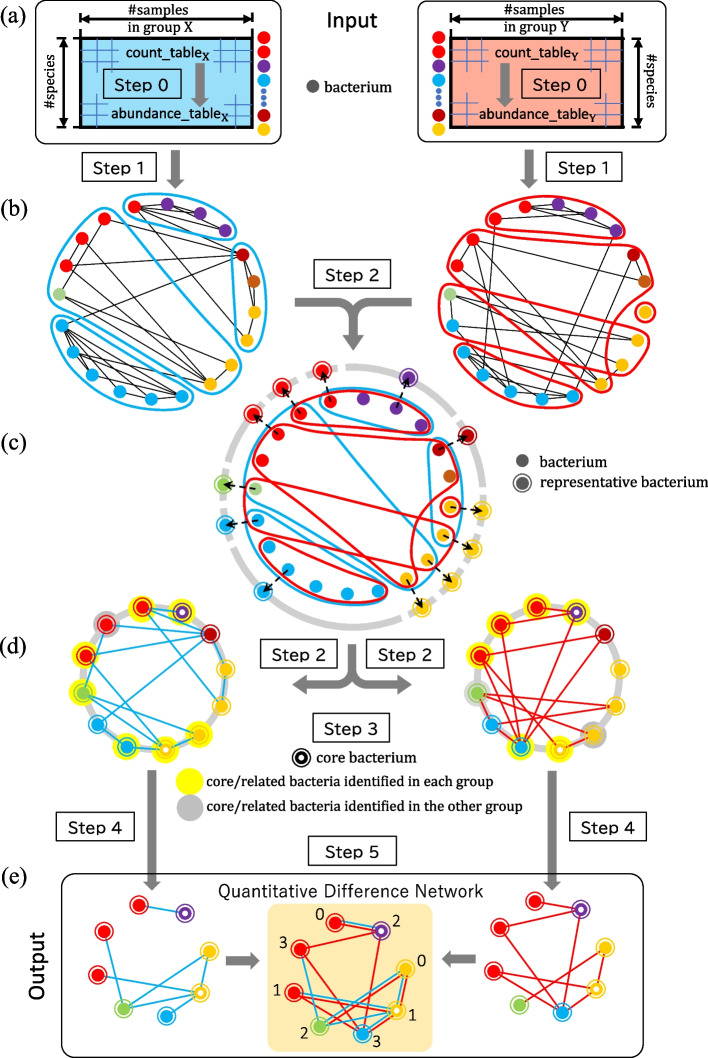
Conceptual diagram of the process performed by the method proposed in this paper. **a** The input is the data of two groups X and Y representing the abundance of bacteria. In this figure, we assume that there are 20 kinds of bacteria (indicated by the filled-in circles), and distinguish the classification of each bacteria by phylogenetic annotation using colors (6 colors in this figure). The abundance of each bacteria for each sample is originally given as a count (count_table), which is normalized to a relative abundance (abundance_table) in Step 0. **b** In Step 1, a bacterial correlation network based on the co-occurrence of bacteria is constructed for each group, by removing noise using existing tools based on the relative abundance of bacteria. In Step 2, in order to eliminate noise due to false correlations, we perform clustering for each of the two groups to unify (contract) the same bacterial groups, and unify the bacterial groups whose phylogenetic annotation classification matches within the same cluster. **c** Then, for each unified group, the bacteria with the largest average abundance among all the bacteria in that unified group is selected as the representative bacteria of the unified group. The process of contraction to remove false correlations up to this point is the first feature of the proposed method. **d** Then, in Step 3, the bacteria that significantly increase in group Y compared to group X among the representative bacteria are identified as the core bacteria. In Step 4, a network consisting of core bacteria and their related bacteria is constructed. In the figure, the core bacteria and their related bacteria in the group are shown in yellow background circles, and those in the other group are shown in gray background circles. Their combined network is the final network. **e** In Step 5, the two networks constructed in Step 4 are compared, and identify bacteria with large QNetDiff scores, which represent the level of rewiring of the links. Then output them together with various statistical values. The process that focuses on rewiring is the second feature of the proposed method

An overview of our proposed method is presented in Fig. 1, and the detail of each step will be discussed in the subsequent subsections.

### Overview

In this subsection, we give an outline of the process performed by the method proposed in this paper. This method first constructs a bacterial correlation network (hereafter this is also referred to simply as “network”) for each group based on the co-occurrence relationship of bacteria, from the counts of bacteria in each fecal sample. Then, in each network, we remove false correlations by unifying the nodes (bacteria). Then, we first identify the bacteria that significantly increase in one group and reconstruct a network consisting of only highly related bacteria and then list the important nodes in such a network. The inputs and outputs of the entire method are given in the following Table 1. Here, the definitions of the rewiring index (QNetDiff score) in the table will be given in the subsequent explanations.

The proposed method carries out the following six steps (Steps 0 to 5) in sequence.

**Step 0**: Conversion of bacterial counts to relative abundance
**Step 1**: Construction of bacterial correlation networks based on bacterial co-occurrence relationships
**Step 2**: Unification of similar bacteria (elimination of false correlations) and selection of representative bacteria
**Step 3**: Selection of core bacteria for bacterial correlation network
**Step 4**: Construction of a network consisting of core bacteria and related bacteria
**Step 5**: Calculation of rewiring index and other features of each bacteria

In Detailed descriptions of processes in each step section, we provide a more detailed step-by-step explanation.

**Table 1:** Specification of inputs and outputs of the proposed method

**Input**	(1) Counts per sample in X and Y of bacteria observed in two groups X and Y.
	(2) Phylogenetic annotation of bacteria*.
	(3) Whether the bacteria of interest is increased in either X or Y (we assume it to be Y, without loss of generality).
**Option**	(a) Threshold value of the correlation coefficient to be used as the edge weights of the network (default value is 0.4).
	(b) Threshold *p* value for testing whether each bacteria increased in the group specified in input (3) (default value is 0.005).
**Output**	The rewiring index (QNetDiff score) of the bacteria that constitute the core of the bacterial correlation network and its associated bacteria, as well as the degree, mean abundance in each group of X and Y, and *p* value of the change from group X to Y.
	(In addition, the list of bacteria that significantly increased from group X to Y, the list of bacteria belonging to each bacterial group unified for the elimination of false correlations, and its representative.)

*Bacterial phylogenetic annotation consists of the levels of phylum $\rightarrow$ class $\rightarrow$ order $\rightarrow$ family $\rightarrow$ genus $\rightarrow$ species $\rightarrow$ strain. In the example of this paper, bacterial names are given at the genus level. This method is applicable if information of two consecutive levels are given

### Detailed descriptions of processes in each step

In this subsection, we describe the process performed in each step in detail. The input data is as follows: The list of all bacteria in input (1), each of which is referred to as a name at some level of phylogenetic annotation, such as genus, species, and so on. The counts of bacteria per sample in the two groups X and Y are given as two-dimensional arrays, respectively, of size [#distinct bacteria name]$\times$[#samples]. As input (2), the name of one level higher in the phylogenetic annotation of the bacteria is given. Also in the subsequent explanations, we assume that Y is specified as input (3) without loss of generality.

#### Step 0: Conversion of bacterial counts to relative abundance

Since the total number of bacteria detected in each sample is different, it is necessary to normalize the counts to relative abundance data for each sample to allow comparisons between samples. Since the same process is applied for two groups, we describe the detailed process only with group X.

We normalize the counts ($\texttt {count}\_\texttt {table}_{\textrm{X}}$) for each sample and obtain their relative abundance ($\texttt {abundance}\_\texttt {table}_{\textrm{X}}$). Specifically, for the *i*th bacteria and *k*th sample, we obtain the relative abundance by the following calculation:

$$\begin{aligned} \begin{array}{l} \texttt {abundance}\_\texttt {table}_{\textrm{X}}[\texttt {bacteria}[i]][\texttt {sample}[k]] \\ \displaystyle { \hspace{2cm} = \frac{ \texttt {count}\_\texttt {table}_{\textrm{X}}[\texttt {bacteria}[i]][\texttt {sample}[k]]}{\sum _{h=1}^{|\texttt {bacteria}|}{} \texttt {count}\_\texttt {table}_{\textrm{X}}[\texttt {bacteria}[h]][\texttt {sample}[k]]} } \end{array} \end{aligned}$$

where the count of the *i*th bacteria (in the list bacteria of all bacteria) in the *k*th sample of group X (in the list sample of group X samples) is expressed by count_table_X_[bacteria[*i*]][sample[*k*]].

If the label “unclassifed” is included in bacteria, it is removed from bacteria after the above calculation. This process may result in the sum of the relative abundances for each sample not being equal to 1.

#### Step 1: Construction of bacterial correlation networks based on bacterial co-occurrence relationships

In this step, correlation coefficients based on the co-occurrence relation between all bacteria are calculated for each of the two groups X and Y, and two networks are constructed using them as edge weights. To calculate correlation coefficients between bacteria, we use the tool called SparCC [20], which is a tool for calculating the correlation among all bacteria based on the counts of each bacteria. SparCC is able to calculate correlation coefficients with eliminating the incorrect correlations caused by bacteria that are present only in small numbers. The SparCC used in this study is an improved version, which is known as SparCC3 [17].

By giving a two-dimensional array $\texttt {count}\_\texttt {table}_{\textrm{X}}$ of counts of group X as an input to SparCC, we obtain as output a two-dimensional array $\texttt {correlation}_{\textrm{X}}$ of correlation coefficients of size [#distinct bacteria]$\times$[#distinct bacteria]. For the coefficient table $\texttt {correlation}_{\textrm{X}}$ obtained in this way, if each element is greater than the designated threshold (edge_threshold), the value is taken as the weight of the corresponding edge of the network (element of the adjacency matrix); otherwise, that is if it is less than or equal to the threshold value, the value of the element of the adjacency matrix corresponding to it is set to 0. By the above process, we obtain two bacterial correlation networks (their adjacency matrices) corresponding to groups X and Y, respectively. Here, the default value of edge_threshold is set to the standard 0.4.

Next, isolated nodes in both networks are removed from the network. For this, we first remove them from the list of bacteria (bacteria) to obtain the list of effective bacteria (bacteria_effective). Then we prepare the adjacency matrix whose rows and columns consist only of those in bacteria_effective. That is, it is a two-dimensional array ($\texttt {correlation}\_\texttt {effective}_{\textrm{X}(\textrm{Y})}$) whose (*i*, *j*)-element is the edge weight between the nodes corresponding to bacteria_effective[*i*] and bacteria_effective[*j*] in the group X(Y).

We show a pseudo-code of the process of this step in Listing 1 in Additional file 1: Section 1.1.

#### Step 2: Unification of similar bacteria (elimination of false correlations) and selection of representative bacteria

In metagenome analysis, a DNA read may map to multiple closely related bacterial genomes with the almost same score. In such a case, there could happen a false correlation in which the correlation between closely related bacteria appears much higher than its actual state due to the similarity in the abundance of the closely related bacteria. Therefore, in order to prevent such illegal correlations from making actually meaningless bacteria (nodes) appear to be important bacteria by having large degrees on the network, we remove the illegal edges by unifying such groups of closely related bacteria. On the network, this is achieved by *clustering* of nodes corresponding to such bacteria and replacing multiple nodes corresponding to closely related bacteria with a single representative node (i.e., *contraction*). Specifically, clustering of nodes is done for each of the two networks obtained in Step 1, and bacterial clusters are collapsed such that the corresponding nodes belong to the same classification at one level higher (i.e., species for strain, genus for species, etc.) of the bacterial name in the phylogenetic annotation of input (2) and the corresponding nodes belong to the same cluster in each of the two groups.

In the following, we will explain the process of this Step 2 process in more detailed three steps (2.1 to 2.3).

*Step 2.1. Identification of bacteria that may be affected by noise (clustering of nodes)*

We perform clustering of nodes using the Louvain method [21], which is known as a general clustering method applicable to weighted networks. Due to the randomness of the Louvain method, we apply it with fixed random seed values by default. For actual calculations, we use the python-louvain library.

By giving the adjacency matrix ($\texttt {correlation}\_\texttt {effective}_{\textrm{X}}$) as an input to the Louvain method, we obtain an array ($\texttt {cluster}_{\textrm{X}}$) whose elements are the cluster numbers of each node as an output. For example, when the cluster number of bacteria_effective[*i*] in group X is 1, then $\texttt {cluster}_{\textrm{X}}[\texttt {bacteria}\_\texttt {effective}[i]]=1$.

*Step 2.2. Determination of the group of bacteria to be unified (determination of the set of nodes to be contracted)*

In whatever sample groups, the false correlations considered by this method occur between closely related bacteria. Therefore, we consider as the target of unification (contraction) maximal sets of bacteria (nodes) that belong to the same phylogenetic category at one level higher, and that have a correlation (edge) with each other in both of the two groups.

Specifically, for a pair of bacteria bacteria_effective[*i*] and bacteria_effective[*j*], we consider the following two conditions:

1. The two bacteria belong to the same phylogenetic category at one level higher. This is determined by
  $$\begin{aligned} \texttt {sup}\_\texttt {category}[\texttt {bacteria}\_\texttt {effective}[i]] = \texttt {sup}\_\texttt {category}[\texttt {bacteria}\_\texttt {effective}[j]]. \end{aligned}$$
2. Those two bacteria belong to the same cluster in each of the two groups in the clustering results of Step 2.1. This is determined by
  $$\begin{aligned} \begin{array}{c} \texttt {cluster}_{\textrm{X}}[\texttt {bacteria}\_\texttt {effective}[i]] = \texttt {cluster}_{\textrm{X}}[\texttt {bacteria}\_\texttt {effective}[j]]\ \ \text{ and }\ \\ \ \texttt {cluster}_{\textrm{Y}}[\texttt {bacteria}\_\texttt {effective}[i]] = \texttt {cluster}_{\textrm{Y}}[\texttt {bacteria}\_\texttt {effective}[j]]. \ \ \ \ \ \ \ \ \end{array} \end{aligned}$$

We call a (sub)set of bacteria any pair of whose element (bacteria) satisfy the above two conditions a *similar bacteria group within a cluster*. In this step, bacteria that are in the same cluster as a clustering results in Step 2.1 and belong to the same phylogenetic category at one level higher are identified. The output in this step is an array (bacteria_groups) containing similar bacteria groups within a cluster.

We show a pseudo-code of the process of this step in Listing 2 in Additional file 1: Section 1.2.

*Step 2.3. Unification of similar bacteria within a cluster (contraction of similar node sets)*

For each similar bacteria group within a cluster in the array bacteria_groups, we select the bacteria with the highest average abundance in all samples in that group (e.g., for the *i*th bacteria, $\frac{1}{N} \sum _{\ell =1}^N\texttt {abundance}\_\texttt {table}[\texttt {bacteria}[i]][\texttt {sample}[\ell ]]$, where *N* is the number of samples), and we call it the *representative bacteria* of the similar bacteria group within the cluster. These representative bacteria are the elements of the array bacteria_representative as the nodes of the network after unifying similar bacteria groups within the cluster. Accordingly, the adjacency matrices of the networks after unification of similar bacteria groups within a cluster is $\texttt {correlation}\_\texttt {representative}_{\textrm{X}}$ and $\texttt {correlation}\_\texttt {representative}_{\textrm{Y}}$, and the sizes of the rows and columns are equal to the size of bacteria_representative.

Since the same process is applied for two groups, we describe the detailed process only with group X. The two-dimensional array

$\texttt {correlation}\_\texttt {representative}_{\textrm{X}}[\texttt {bacteria}\_\texttt {representative}[i]][\texttt {bacteria}\_\texttt {representative}[j]]$ (i.e., the edge weights between bacteria_representative[*i*] and bacteria_representative[*j*] in the network of group X after contraction) is computed as the average weight of all the edges between two similar bacteria groups within a cluster to which the bacteria bacteria_representative[*i*] and bacteria_representative[*j*] belong, respectively (or 0 if there is no edge).

We show a pseudo-code of the process of this step in Listing 3 in Additional file 1: Section 1.2.

#### Step 3: Selection of core bacteria for bacterial correlation network

In this step, we select the core bacteria of the bacterial correlation network. The core bacteria are ones that satisfy the representative bacteria obtained in Step 2, and also significantly increased in the focused group (designated by input (3)). For this purpose, we use one-sided Mann–Whitney’s U test [22] to determine the *p* value for each representative bacteria, if its abundance is increased in group Y compared to group X.

The array bacteria_core consisting of representative bacteria whose *p* values obtained by the test satisfying $p<$ p_threshold is the output of this step. The default value of p_threshold is set to 0.005, which is a commonly used significance level. Those bacteria, which are representative bacteria in the network whose false correlations are removed by unifying similar bacteria groups within clusters in Step 2, and which are determined to be significantly increased bacteria in Step 3, are called *core bacteria*.

We show the input and output of this step in Additional file 1: Section 1.3.

#### Step 4: Construction of a network consisting of core bacteria and related bacteria

In this step, we obtain output (1) by extracting the core bacteria and their related bacteria (neighboring nodes) that may have different properties from those of the core bacteria but are correlated with them.

Let *V* be the set of nodes corresponding to the representative bacteria in the bacteria_core obtained in Step 3 and the set of nodes adjacent to one of those nodes in at least one of the two networks after the contraction (unification of similar bacteria groups in a cluster), and let $E_{\textrm{X}}$ be the set of edges in the network after the contraction and both of whose end nodes belong to *V*. The network is constructed by letting $G_{\textrm{X}} = (V, E_{\textrm{X}})$. These $G_{\textrm{X}}$ and $G_{\textrm{Y}}$ obtained in this way become part of the output. (Visualizations of these networks are also provided using the NetworkX [23] library.)

We show a pseudo-code of the process of this step in Listing 4 in Additional file 1: Section 1.4.

#### Step 5: Calculation of rewiring index and other features of each bacteria

In this step, several features of each bacteria are computed by using the network constructed in Step 4.

We calculate the *rewiring index* (QNetDiff score), which is newly proposed in this paper as a measure of the level of rewiring of bacteria between two networks, of each node as a feature of each bacteria on the network after contracting the nodes corresponding to similar bacteria within the cluster. For this purpose, we define below the rewiring index $\texttt {QNetDiff}_{G_{\textrm{X}}, G_{\textrm{Y}}}[v]$ of node *v* in the two networks $G_{\textrm{X}}$ and $G_{\textrm{Y}}$ (simply $\texttt {QNetDiff}[v]$ if the two networks $G_{\textrm{X}}$ and $G_{\textrm{Y}}$ to compare are trivial).

The symmetric difference of the set of adjacent nodes in each of the two (unweighted) networks of a node can be regarded as representing the change of the edge incident to that node. For unweighted networks, the level of the rewiring can be regarded simply as the size of the set of the symmetric difference, but we extend this notion to edge-weighted networks. Since the symmetric difference between two sets is their union minus their intersection, we introduce the notion of the size of weighted union and intersection for edge-weighted networks.

Now, let $A_{\textrm{X}}$ and $A_{\textrm{Y}}$ be the (weighted) adjacency matrices of the two networks $G_{\textrm{X}}$ and $G_{\textrm{Y}}$, respectively. Since the intersection can be regarded as the unchanged part of the two networks, we define the size $A_{\textrm{X}\cap \textrm{Y}}[v]$ of the edge-weighted intersection of node *v* as follows:

$$\begin{aligned} A_{\textrm{X}\cap \textrm{Y}}[v]=\sum _{w\in N(v)}\min \bigl \{A_{\textrm{X}}[v][w], A_{\textrm{Y}}[v][w]\bigr \}. \end{aligned}$$

Similarly, since the union can be regarded as a part contained in at least one of the two networks, we define the size $A_{\textrm{X}\cup \textrm{Y}}[v]$ of the edge-weighted union of node *v* as follows:

$$\begin{aligned} A_{\textrm{X}\cup \textrm{Y}}[v]=\sum _{w\in N(v)}\max \bigl \{A_{\textrm{X}}[v][w], A_{\textrm{Y}}[v][w]\bigr \}. \end{aligned}$$

Using these, we define the size of the edge-weighted symmetric difference $A_{\textrm{X}\triangle \textrm{Y}}[v]$ as

$$\begin{aligned} A_{\textrm{X}\triangle \textrm{Y}}[v]=A_{\textrm{X}\cup \textrm{Y}}[v]-A_{\textrm{X}\cap \textrm{Y}}[v], \end{aligned}$$

and this is the rewiring index $\texttt {QNetDiff}[v]$ of node *v*.

In addition to the QNetDiff scores computed for each node (bacteria) according to this formula, the entire output of this method includes the features of each node such as the degree and average abundance in the respective network, and *p* value of the change from group X to Y.

We show a pseudo-code of the process of this step in Listing 5 in Additional file 1: Section 1.5.

### Application to human gut microbiome data

In the next section, we show an example of applying the proposed method to actual data. The data to be applied are the counts of 2001 human gut bacterial genera estimated from the number of gene sequence fragments obtained by 16SrRNA gene amplicon analysis for each stool sample of 576 patients, including colorectal cancer patients [18]. (We remark here that this is publicly available open access data and there is no direct involvement of any patients in this study.) Bacterial names are given at the level of genus, and are classified at the level of family (one level higher) according to phylogenetic annotation. Each sample is classified into one of five groups (Healthy (251 sample size), Multiple_polyps (67), Stage_0 (73), Stage_I_II (111), Stage_III_IV (74)) as the disease progress level from healthy to worse by medical diagnosis. In this example, we let Healthy and Stage_0 be the groups X and Y, respectively. Specifically, we apply the proposed method to the data of the counts of the two groups Healthy and Stage_0 as input (1) in Overview section. In the following, some of the results are shown in the order of the steps indicated in Overview and Detailed descriptions of processes in each step sections. Note that in the input (3) (which of the two groups to focus on) of Overview section, Stage_0 is specified (as group Y), and all options are assumed to be default values. In order to validate the proposed method, a two-group comparison with Healthy (up to Step 3 of the proposed method) is also performed on the data of stages other than Stage_0 by using the existing method, and clarify which bacteria are significantly increased. The method proposed in this paper, that is, a method, which is based on network analysis, for finding bacteria with characteristic behavior between two groups from the viewpoint of rewiring, is implemented and provided as a usable tool (https://github.com/DiscreteAlgorithms/QNetDiff).

## Results

In this section, we illustrate an example of applying the method proposed to actual data [18] of colorectal cancer patients. Here, we assume that group X = Healthy (healthy group) and group Y = Stage_0 (diseased group). As a result, we confirm that bacteria that cannot be detected simply by their abundance or comparison of their abundance are identified as characteristic bacteria in the bacterial correlation network.

*Step 1* In the originally constructed bacterial correlation networks of the healthy and diseased groups, both of which consists of 2001 genera, there are 1743 genera that do not have any correlation (edges) with other genera (nodes) in any of the two groups. After removing them from the total of 2001 genera, the number of effective bacteria (i.e., nodes), constituting the bacterial correlation network output by this step becomes 258. The number of edges was 1372 for group X (Healthy) and 1747 for group Y (Stage_0), and the sum of the edge weights, which is the total correlation coefficient between pairs of genus, was 758.524 for group X (Healthy) and 952.879 for group Y (Stage_0). Similar information about other groups are presented in Supplementary Table 1 in Additional file 1: Section 2.1.

*Step 2* Clustering of the bacterial correlation networks of the healthy and diseased groups showed that the 258 effective bacteria were divided into 134 clusters of similar genera. Among those, 102 consisted of a single genus and 32 were similar groups of two or more genera. The unification of similar bacteria within a cluster (i.e., contraction of the set of similar nodes) is practically performed for bacterial groups consisting of more than two kinds of bacteria. This results the number of nodes in the bacterial correlation network to 134 after unification. Detailed information is given in Supplementary Table 2 in Additional file 1: Section 2.1

The size of the largest unified group of the genera was 35, and its representative bacteria was *Shigella*, whose sup_category was *Enterobacteriaceae*. A complete list of bacterial groups and representative bacteria in the unified clusters is shown in Supplementary Table 3 in Additional file 1: Section 2.2. We also show a list of bacteria unified in a single genus in Supplementary Table 4 in Additional file 1: Section 2.2.

**Fig. 2:**
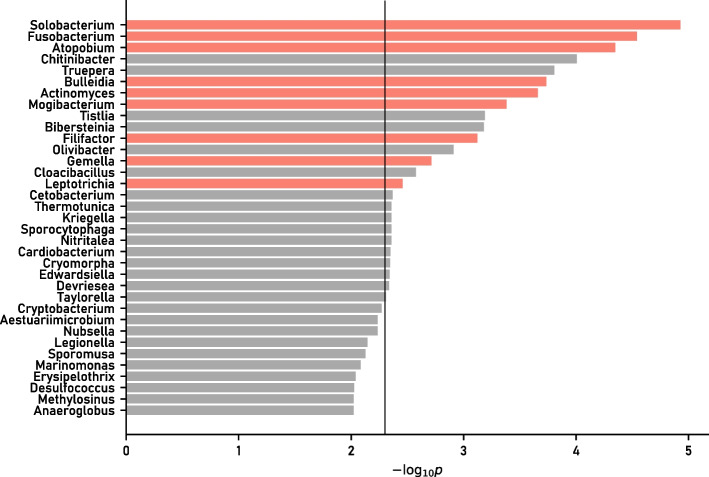
All genera with *p* values below 0.01 among all 2001 genera and their $-\log _{10}p$ are shown. The black vertical line indicates the significance level $p=0.005$. The genera shown in red represent the genera that remain as representative bacteria after the unification of similar bacteria within the cluster in Step 2, and are selected as core bacteria after being tested as significantly increasing ones in Step 3. The genera shown in gray represent the genera that are significantly increased in the comparison of two groups but do not correlate with other genera or are not representative bacteria

*Step 3* Among all 2001 bacteria genera included in the input, the genus whose *p* values are below 0.01 by the test, and those negative values after taking their ordinary logarithm ($-\log _{10}p$) are shown in Fig. 2. There are already only 25 genera with *p* values below 0.01. Among those, nine of them (shown in red) are found to be representative bacteria.

**Fig. 3:**
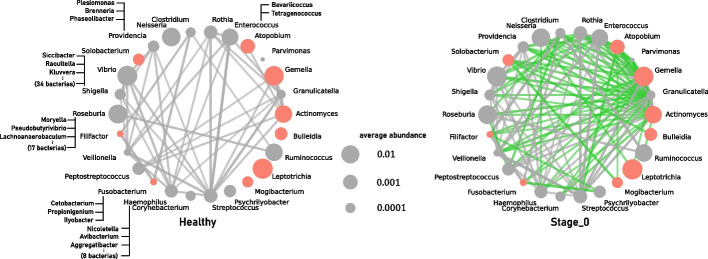
Bacterial correlation networks consisting of core bacteria and their related bacteria for group X (Healthy) (left) and group Y (Stage_0) (right). Red nodes represent core bacteria, and gray nodes are genera that show a correlation of 0.4 or more with one or more core bacteria in any of the groups. Green edges represent pairs of genera that are not correlated in the other group, and gray edges represent those that are correlated in both groups. Each pair of genera is depicted as a thicker and denser edge when the correlation (branch weight) is greater. The size (area) of a node represents the relative abundance of the corresponding bacteria

*Step 4* Based on the core bacteria identified in Steps 2 and 3, we construct a network consisting of the core bacteria and their related bacteria for the healthy and diseased groups, respectively. Figure 3 shows those networks. The red nodes represent the core bacteria. The green edges represent bacteria pairs that do not correlate in the other group, and in the present data, such a pair exists only in group Y (Stage_0). Each pair of genus is drawn as a thicker and darker edge when it has a higher weight (correlation coefficient).

**Fig. 4:**
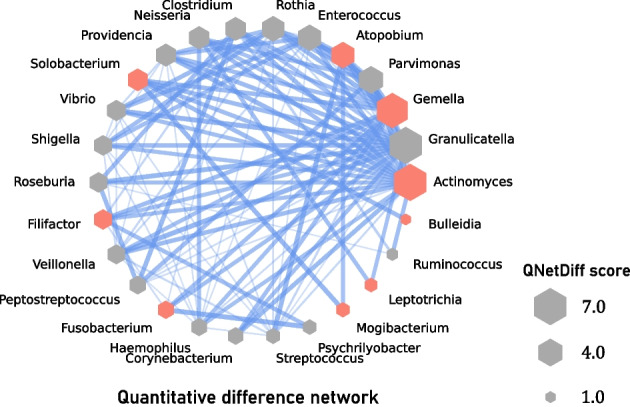
A network whose edge weights are the difference of those for the networks of Healthy and Stage_0, and the values are expressed as the thickness and density of the edges (the weight is 0 when there is no edge). The size (area) of the node represents the QNetDiff score. Nodes are arranged in counterclockwise order of QNetDiff score, and *Actinomyces* is the bacteria with the largest QNetDiff score. *Granulicatella* has been reported to form a biofilm in the oral cavity together with *Fusobacterium*, which is known to increase with the progression of colorectal cancer

*Step 5* We show in Fig. 4 a visualization of the network by taking the difference of the edge weights for the two networks consisting of the core bacteria and their related bacteria constructed in the Step 4. In this network, the sum of the weight of the edges incident to a bacteria is equal to the QNetDiff score of the bacteria, and its size is shown as the size (area) of the hexagon in Fig. 4. We can see that *Actinomyces*, *Granulicatella*, *Gemella*, etc. show relatively high QNetDiff scores.

**Fig. 5:**
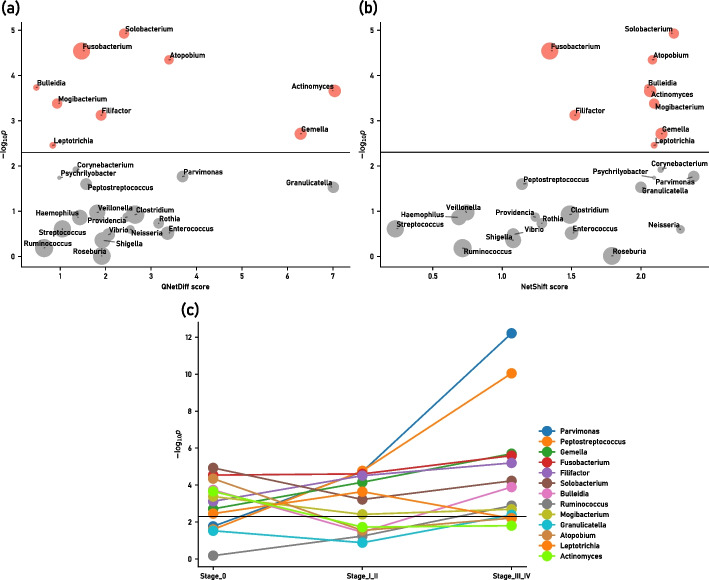
Relationship between **a** QNetDiff scores (horizontal axis) for all 26 core bacteria and their related bacteria, and $-\log _{10}p$ (vertical axis) for the significant difference *p* obtained from the test comparing two groups, Healthy and Stage_0; **b** similarly, the relationship between NetShift score (horizontal axis) and $-\log _{10}p$ (vertical axis). The black horizontal line indicates the significance level $p=0.005$, and the size of the circle (area) indicates the mean abundance of each genus in the total samples. **c** The value $-\log _{10}p$ of the significant difference *p* obtained by the two-group test for the increase of abundance in each of the three stages (Stage_0, Stage_I_II, Stage_III_IV) compared to Healthy. (Here, only genera whose *p*-values are lower than the significance level at any of the stages are shown. The black horizontal line indicates the significance level of $p=0.005$)

Finally, we show in Fig. 5a a scatter plot of the relationship between the QNetDiff score and $-\log _{10}p$ for each bacteria in the network consisting of core bacteria and their related bacteria. We also show in Fig. 5b a similar scatter plot about NetShift score, for the comparison with other tools in Subseciton . Figure 5c shows a one-tailed test to determine if the abundance of each genus increased also for Stage_I_II and Stage_III_IV compared to Healthy, and three *p* values obtained for the genus whose abundance is below the significance level of $p=0.005$ in any of the stages, including Stage_0, are shown as line graphs. We also give the QNetDiff scores for all 26 representative bacteria, the degree in each network for each genus, the average abundance, and the *p* values for the change from Healthy to Stage_0 in Supplementary Table 5 in Additional file 1: Section 2.3.

## Discussion

In this paper, we develop and provide a new method for finding the difference between two groups of data that generally indicate the abundance of bacteria. Specifically, unlike existing methods that are based only on fundamental analysis such as the amount of each bacteria and its increase or decrease, this method detects and quantifies rewiring, which is only revealed through network analysis by focusing on advanced data analysis, such as the correlation among bacteria. Then, we applied the proposed method to two groups of human colorectal cancer patients, the healthy group and the diseased group, and the bacteria present in their stool specimens. In this section, we discuss the significance and effectiveness of the proposed method based on the results of its application.

### Features and novelty of the proposed method

One of the features of the proposed method is that it focuses on the phenomenon called rewiring, in which certain bacteria change their co-occurrence relationship with other bacteria as the disease progresses, and provides a method to quantify this phenomenon by introducing an indicator that expresses the level of rewiring of each bacteria. Attempts to quantify rewiring based on bacterial correlation networks are not necessarily new, however, this paper newly provides a definition for the level of rewiring based on the (weighted) symmetric difference of edges incident to the nodes corresponding to bacteria. As a result, as we will see in the next subsection (Detailed descriptions of processes in each step section), we have succeeded in finding bacteria that are difficult to detect by existing methods, although they are the characteristic bacteria associated with rewiring and have differences between two groups. In particular, a feature of the proposed QNetDiff score is that it is more sharpened in comparison with other methods, and thus it has an ability to detect bacteria with extreme features more clearly and easily.

One of the major issues in conducting such metagenomic analyses based on networks is the noise generated in the construction of bacterial correlation networks. Typically, it is caused by an imbalance in the abundance of bacteria for which correlation is calculated, however, tools to reduce this kind of noise, such as SparCC [17], are available. On the other hand, a single read may map to multiple closely related bacterial genome, as the genomic region to be mapped are almost identical in sequence. When correlations are obtained for such data, there is a problem of false correlation, in which the correlations of closely related bacteria are much higher than they actually are, but this is unavoidable for such closely related bacteria, and no effective way to deal with this noise was known.

To cope with this problem, our method suggests and employs a new noise reduction method for the construction of bacterial correlation networks. Specifically, we first construct a bacterial correlation network and then cluster the bacteria (using the Louvain method, which is a typical clustering method). Then, we utilize phylogenetic annotation to unify (contract) bacteria belonging to the same classification at one level higher than the bacterial name, which leads to removing false correlations (edges representing false correlations). For the bacteria representing the unified bacterial group, the bacteria with the largest average abundance in all the samples in the group are selected.

### Confirmation of the effectiveness of the proposed method

In Results section, we constructed a bacterial correlation network for human colorectal cancer data [18] with healthy group (Healthy) and diseased group (Stage_0) as two groups and applied this tool to actual cases. The results are shown in Figs. 2, 3, 4, and . In particular, according to the scatter plot in Fig. 5a which shows the relationship between the QNetDiff score and $-\log _{10}p$ for each bacteria, we can see that *Granulicatella* and *Parvimonas*, which are located mainly in the lower right region and have the second and fourth highest QNetDiff scores, respectively, are found to have large QNetDiff score although the *p* value does not reach the significance level, that is, they can be found as genera that are significantly rewiring in the bacterial correlation network although the change in the abundance of the two groups is not necessarily large.

Among these genera, *Granulicatella* has been reported to form a biofilm in the oral cavity together with *Fusobacterium*, which is known to increase with the progression of colorectal cancer [24], and although the *p* value did not reach the significance level, it may affect the disease in the intestine together with other bacteria. Also, *Parvimonas* is a genus whose *p* value does not reach the significance level in Stage_0, the early stage of colorectal cancer, but increases further in the later stages of colorectal cancer. As observed, we can see that the proposed method reliably identifies in the early stages bacteria with high QNetDiff scores as those with strong functional associations with bacteria associated with colorectal cancer by in vitro experiments, and bacteria that have been reported to be associated with colorectal cancer by multiple cohort studies [2, 3].

A genus with similar characteristics to *Parvimonas* is *Gemella*, which has the third highest QNetDiff score. This genus has already significantly increased at Stage_0, but we can see in Fig. 5c that it increases further in later stages. In this way, QNetDiff score may detect genera that increase further in later stages than the stage adopted as the disease group, and it is assumed that the score may be used to predict disease-associated bacteria. These results suggest that QNetDiff score proposed in this paper to represent the level of rewiring is also useful in more advanced analyses of bacterial correlation networks.

### Comparison with other tools

There are a limited number of existing methods that use network analysis for bacterial correlation networks, such as the one employed in this paper, for the purpose of comparing two groups of data and clarifying the differences between them. One of the few such methods is NetShift [25]. In this subsection, we apply NetShift to a bacterial correlation network consisting of core bacteria and their related bacteria constructed from the data on human colorectal cancer to which our proposed method was applied in Results section. Then we compare our QNetDiff, our proposed method in this paper, and NetShift.

As already explained in Results section, Fig. 5a shows the relationship between the QNetDiff scores of all 26 bacteria that constitutes the bacterial correlation network of the core bacteria and their related bacteria and $-\log _{10}p$ for the significant difference *p* obtained from the test comparing the two groups, Healthy and Stage_0. Similarly, Fig. 5b shows the relationship with the NetShift scores (that are shown in Supplementary Table 5 in Additional file 1: Section 2.3 as well).

From the comparison between Fig. 5a and b, the genera such as *Granulicatella*, *Actiomyces*, *Gemella*, and *Parvimonas*, which show high values in QNetDiff score, also show high values at some level in NetShift score. On the other hand, however, NetShift shows the same level of scores for relatively many genera other than the above, which makes it difficult to understand how only *Granulicatella*, *Actiomyces*, and *Gemella* differ from the others. In other words, we can see that NetShift provides a large number of candidates, while QNetDiff can focus on more sharpened and important candidates. From these observations, it is assumed that QNetDiff is suitable for searching bacteria with stronger distinctive propensity between two groups, and NetShift could be used when we want to select a larger number of bacteria as candidates, although the possibility of including noise is higher.

### Limitation

The proposed method may have performance limitations or restrictions in the following points.

- False correlations (edges of bacterial correlation networks) are effectively eliminated by the unification (contraction) of similar bacteria, which is a characteristic feature of the proposed method. On the other hand, we cannot deny the possibility that edges due to positive correlation may also be eliminated. Therefore, the tool implementing this method has an option to skip this contraction process. In addition, output (3) provides information on what bacteria have been centralized to the representative bacteria, and therefore, it is possible to check the unification process and to examine its validity qualitatively.
- It is difficult to determine the threshold for the correlation coefficient between bacteria. We set the default threshold value to be 0.4. However, it is confirmed that if we set it smaller (larger), then more (less) edges and nodes remain in the bacterial correlation network. Therefore, more (less) candidate bacteria appear. For this point, we think that the developed tools allow users to arbitrarily change the correlation threshold that serves as a criterion for putting edges among bacteria when constructing a bacterial correlation network, and that such a function can be used to adjust and cope with this issue to some extent.
- As a criterion for selecting representative bacteria from the unified bacterial groups, we currently adopt the maximum average value of relative abundance. In this case, there is a possibility that some biologically important bacteria could be hidden by unification. Therefore, in the developed tool, a list of unified bacteria is presented as output (3), together with their representatives. We can address this issue by checking the structure of the clusters.

## Conclusion

In this paper, we propose a method for finding candidate bacteria associated with changes in the attribute values of two sets of samples, which have different attribute values mainly related to some human diseases (typically, a healthy group and a diseased group), by focusing on the phenomenon called rewiring of the bacterial correlation network. The proposed method was applied to actual data of human colorectal cancer patients, and we verified its usefulness.

Although this method was developed for human diseases with the identification of their causative bacteria as a starting point, it goes without saying that the scope of its application is not limited to this. In other words, it can be widely applied when the elements in each of two groups of data with different attribute values are correlated in some way, and the differences between the two groups can be detected based on the correlations (networks). Furthermore, it is expected to be useful not only for the purpose of comparing two groups but also for interpreting the meaning of the data of each group.

### Supplementary Information

**Additional file 1**. Supplementary Materials.

## Data Availability

The source code for QNetDiff algoritms and data analysis scripts can be accessed from GitHub (https://github.com/DiscreteAlgorithms/QNetDiff). The data of large intestinal cancer patients for the application of QNetDiff are available from DDBJ Sequence Read Archive (DRA): DRA006684 and DRA008156. (urlhttps://ddbj.nig.ac.jp/DRASearch/submission?acc=DRA006684 and https://ddbj.nig.ac.jp/DRASearch/submission?acc=DRA008156, last accessed Jan. 31, 2021.
