# Supplementary material for: QNetDiff: a quantitative measurement of network rewiring
Source: BMC Bioinformatics. 2024 Mar 18;25:118. doi: 10.1186/s12859-024-05702-z (PMC10946107; doi:10.1186/s12859-024-05702-z)
Supplement: Supplementary file 1 — Additional file 1. Supplementary Materials. [file 12859_2024_5702_MOESM1_ESM.pdf]

Supplementary Materials

for the submission of

QNetDiff: A Quantitative Measurement of Network Rewiring

Shota Nose, Hirotsugu Shiroma, Takuji Yamada, Yushi Uno

1 Pseudo-codes for Some Steps

1.1 Step 1

Input: counts per sample of bacteria in X and Y ($\texttt{count\_table}_X$, $\texttt{count\_table}_Y$).
Output: list of effective bacteria and its adjacency matrix
($\texttt{bacteria\_effective}$, $\texttt{correlation\_effective}_X$, $\texttt{correlation\_effective}_Y$).

Listing 1: Pseudo-code of Step 1.

```
function Step1( count_table_X, count_table_Y, edge_threshold ):
    correlation_X, correlation_Y = SparCC(count_table_X, count_table_Y)

    bacteria_effective = []

    for b1 in bacteria :
        flag = False
        for b2 in bacteria :
            if correlation_X[b1][b2] > edge_threshold or
               correlation_Y[b1][b2] > edge_threshold:
                flag = True
        if flag == True :
            bacteria_effective.add( b1 )

    for b1 in bacteria :
        for b2 in bacteria :
            for S in {X, Y}:
                if correlation[b1][b2] <= edge_threshold:
                    correlation_effective_S[b1][b2] = 0
                else:
                    correlation_effective_S[b1][b2] =
                                        correlation_S[b1][b2]

    return bacteria_effective, correlation_effective_X, correlation_effective_Y
```

1.2 Step 2

Input: list of effective bacteria and its adjacency matrix output by Step 1 (bacteria_effective, correlation_effective$_X$, correlation_effective$_Y$).
Output: list of representative bacteria (bacteria_representative) and adjacency matrix of network after contraction (correlation_representative$_X$, correlation_representative$_Y$).

Step 2.1

Input: list of effective bacteria and its adjacency matrix output by Step 1 (bacteria_effective, correlation_effective$_X$, correlation_effective$_Y$)
Output: cluster number of effective bacteria (cluster$_X$, cluster$_Y$).

Step 2.2

Input: list of effective bacteria output by Step 1 (bacteria_effective) cluster number of effective bacteria (cluster$_X$, cluster$_Y$), one level higher category of bacteria (sup_category).
Output: array of similar bacteria group within a cluster (similar_groups_in_cluster).

Listing 2: Pseudo-code of Step 2.2.

```
function Step2_2( bacteria_effective, clusterX, clusterY, sup_category):

    for b1 in bacteria_effective:
        for b2 in |bacteria_effective|:
            if union_find.isSame( b1, b2 ):
                continue
            if sup_category[b1]==sup_category[b2] and
            clusterX[b1] == clusterX[b2]
                and
            clusterY[b1] == clusterY[b2]:
               union_find.merge( b1, b2 )

    for i = 0 to union_fing.group_num():
        similar_in_cluster_ses[i] ← empty_vector()
    for b in bacteria_effective:
        similar_groups_in_cluster[ union_find.find_group(b) ].add(b)

    return similar_groups_in_cluster
```

Step 2.3

Input: array of similar bacteria groups within a cluster output by
Step 2.2 (similar_groups_in_cluster),
relative abundance average of bacteria (abundance_average).
adjacency matrix of effective bacteria output by Step 1
(correlation_effective$_X$, correlation_effective$_Y$)
Output: list of representative bacteria (bacteria_representative)
and adjacency matrix of network after contraction
(correlation_representative$_X$, correlation_representative$_Y$).

Listing 3: Pseudo-code of Step 2.3.

```
function Step2_3(similar_groups_in_cluster, abundance_average,
   correlation_effective_X, correlation_effective_Y ):

   bacteria_representative ← empty_vector()
   for group in similar_groups_in_cluster:
      max_abundance_bacteria ← NULL
      max_abundance ← 0
      for bacteria in group:
         if max_abundance < abundance_average[bacteria] :
            max_abundance_bacteria = bacteria
            max_abundance = abundance_average[bacteria]
      bacteria_representative.add( max_abundance_bacteria )
   for S in {X, Y}:
      N ← |similar_groups_in_cluster|
      for i = 0 to N:
         for j = 0 to N:
            edge_num ← 0
            edge_weight_sum ← 0
            for bacteria_1 in similar_groups_in_cluster[i]:
               for bacteria_2 in similar_groups_in_cluster[j]:
                  corr = correlation_effective_S[bacteria1][bacteria2]
                  if corr > edge_threshold :
                     edge_num += 1
                     edge_weight_sum += corr
            br_i = bacteria_representative[i]
            br_j = bacteria_representative[j]
            correlation_representative_S[br_i][br_j] = edge_weight_sum / edge_num

   return bacteria_representative, correlation_representative_S
```

1.3 Step 3

Input: relative abundance of bacteria
(abundance_table$_X$, abundance_table$_Y$).
Output: array of core bacteria (bacteria_core).

1.4 Step 4

Input: array of core bacteria output by Step 3 (`bacteria_focused`), array of representative bacteria and its adjacency matrix output by Step 2 (`bacteria_representative`, $\texttt{correlation\_representative}_{\mathrm{X}}$ and $\texttt{correlation\_representative}_{\mathrm{Y}}$).
Output: a pair of networks (G_{X}, G_{Y}) consisting of core bacteria and related bacteria.

Listing 4: Pseudo-code of Step 4.

```
function Step4( bacteria_core, bacteria_representative, correlation_representative
    X, correlation_representativeY ):
    V ← empty_set()
    for b_core in |bacteria_core|:
        V.add( b_core )
        for b_represent in bacteria_representative:
            if correlation_representativeX[b_core][b_represent]
            > edge_threshold or
            correlation_representativeY[b_core][b_represent]
            > edge_threshold:
                V.add( b_represent )

    EX ← empty_set()
    EY ← empty_set()
    for b1 in V:
        for b2 in V:
            edge_weightX = correlation_representativeX[b1][b2]
            edge_weightY = correlation_representativeY[b1][b2]
            if edge_weightX > edge_threshold:
                EX.add( b1, b2, edge_weightX )
            if edge_weightY > edge_threshold:
                EY.add( b1, b2, edge_weightY )
    return (V, EX), (V, EY)
```

1.5 Step 5

Input: a pair of networks (G_{X}, G_{Y}) consisting of core bacteria and related bacteria output by Step 4.
Output: rewiring index (QNetDiff score) `QNetDiff` of core bacteria and related bacteria in G_{X} and G_{Y}.

Listing 5: Pseudo-code of Step 5.

```
function Step5( GX, GY ):
    V, EX ← GX
    V, EY ← GY

    QNetDiff ← hash_map()
    for v in V:
        QNetDiff[v] = 0

    v_tuple_to_weightX ← hash_map()
    for (v1, v2, weight) in EX:
```

```
            v_tuple_to_weight_X[(v1, v2)] = weight
            if v1,v2 not in E_Y
                QNetDiff[v1] += weight
                QNetDiff[v2] += weight

        for (v1, v2, weight) in E_Y:
            if v1, v2 in E_X :
                abs_diff = | weight - v_tuple_to_weight_X[(v1, v2)] |
            else:
                abs_diff = weight
            QNetDiff[v1] += abs_diff
            QNetDiff[v2] += abs_diff
        return QNetDiff
```

2 Supplementary Experiments and Result Data

2.1 Basic Information on Data of Colorectal Cancer Patients used in the Experiments and the Bacterial Correlation Network

Among the 2,001 intestinal genera that appeared in the stool samples of 576 colorectal cancer patients used in this paper, there were 1,717 kinds that did not correlate with other genera in any of the following stages: Healthy, Multiple_polyps, Stage_0, Stage_I_II, Stage_III_IV. Therefore, there are 284 genera that are correlated with other bacteria in one or more of the five stages. In the following Supplementary Tables 1 and 2, we show information on the bacterial correlation network constructed from these genera before and after the unification (contraction) of bacteria. The contraction is performed for Healthy and each of the other four stages, and the results are shown below.

Supplementary Table 1. Basic information on the bacterial correlation network before contraction at each stage.

Stage	#samples	#nodes (bacteria) with edges	#edges	sum of edge weights
Healthy	251	188	1372	758.5
Multiple_polyps	67	199	1617	924.1
Stage_0	73	215	1747	952.9
Stage_I_II	111	182	1313	747.3
Stage_III_IV	74	205	1654	917.7

Supplementary Table 2. Information on the bacterial correlation network after unification of Healthy with each of the other four stages. Stage represents the two groups to be compared, and the unification is performed for the two groups. #nodes is the number of nodes having edges at any stage, and (#nodes having edges) is the number of nodes having edges at each stage.

Stage	#nodes	(#nodes having edges)	#edges	sum of edge weights
Healthy	127	(69)	196	92.7
Multiple_polyps		(81)	248	118.1
Healthy	134	(71)	198	94.2
Stage_0		(100)	339	161.9
Healthy	118	(70)	191	90.1
Stage_I_II		(72)	179	86.7
Healthy	126	(65)	197	93.3
Stage_III_IV		(86)	306	144.7

2.2 List of Genera Groups unified as Similar Bacteria within a Cluster and their Representative Bacteria

We show in Supplementary Table 3 the list of genera groups unified as similar bacteria within a cluster by step 2 of the proposed method and the representative bacteria of each group. Also, Supplementary Table 4 shows a list of bacteria unified in a single genus.

Supplementary Table 3. A list of 32 unified groups of genera (32 groups) of two or more sizes, the size of each group, and the representative bacteria of each group. They contain 156 kinds of genera.

The size of group of genera	Similar bacteria within a cluster (The head **bold** is the representative bacteria)
35	***Shigella***, *Citrobacter, Cronobacter, Dickeya, Enterobacter, Erwinia, Escherichia, Franconibacter, Hafnia, Klebsiella, Klebsiellaquasipneumoniae, Kluyvera, Kosakonia, Lelliottia, Lonsdalea, Mangrovibacter, Obesumbacterium, Pantoea, Pectobacterium, Pluralibacter, Pragia, Pseudocitrobacter, Rahnella, Raoultella, Salmonella, Samsonia, Serratia, Shimwellia, Siccibacter, Sodalis, Tatumella, Trabulsiella, Xenorhabdus, Yersinia, Yokenella*
18	***Roseburia***, *Anaerostipes, Butyrivibrio, Catonella, Coprococcus, Dorea, Hespellia, Lachnoanaerobaculum, Lachnobacterium, Marvinbryantia, Moryella, Murimonas, Oribacterium, Pseudobutyrivibrio, Robinsoniella, Shuttleworthia, Sporobacterium, Stomatobaculum*
13	***Faecalibacterium***, *Acetanaerobacterium, Anaerobacterium, Anaerofilum, Anaerotruncus, Ercella, Ethanoligenens, Hydrogenoanaerobacterium, Intestinimonas, Papillibacter, Pseudobacteroides, Sporobacter, Subdoligranulum*
9	***Haemophilus***, *Actinobacillus, Aggregatibacter, Avibacterium, Mannheimia, Nicoletella, Pasteurella, Phocoenobacter, Vespertiliibacter*
5	***Fusicatenibacter***, *Eisenbergiella, Hungatella, Lactonifactor, Mobilitalea*
5	***Romboutsia***, *Asaccharospora, Intestinibacter, Sarcina, Terrisporobacter*
5	***Slackia***, *Adlercreutzia, Asaccharobacter, Enterorhabdus, Senegalimassilia*
4	***Alkaliphilus***, *Cellulosibacter, Lutispora, Saccharofermentans*
4	***Barnesiella***, *Butyricimonas, Coprobacter, Odoribacter*
4	***Fusobacterium***, *Cetobacterium, Ilyobacter, Propionigenium*
4	***Parabacteroides***, *Macellibacteroides, Microbacter, Tannerella*
4	***Prevotella***, *Alloprevotella, Hallella, Paraprevotella*
4	***Providencia***, *Brenneria, Phaseolibacter, Plesiomonas*

3	***Blautia***, *Natranaerovirga, Vallitalea*
3	***Catenibacterium***, *Erysipelothrix, Kandleria*
3	***Enterococcus***, *Bavariicoccus, Tetragenococcus*
3	***Flavonifractor***, *Anaerovorax, Pseudoflavonifractor*
3	***Massilia***, *Oxalobacter, Pseudoduganella*
3	***Megasphaera***, *Anaeroglobus, Propionispira*
2	***Aeromonas***, *Zobellella*
2	***Alistipes***, *Mucinivorans*
2	***Bilophila***, *Lawsonia*
2	***Brackiella***, *Paenalcaligenes*
2	***Collinsella***, *Coriobacterium*
2	***Eggerthella***, *Gordonibacter*
2	***Holdemanella***, *Faecalitalea*
2	***Megamonas***, *Pectinatus*
2	***Mitsuokella***, *Selenomonas*
2	***Propionispora***, *Anaerosinus*
2	***Roseateles***, *Schlegelella*

Supplementary Table 4. List of bacteria unified in a single genus. There are 92 kinds of genera belonging to this list. These are automatically representative bacteria.

Zoogloea, Zhongshania, Zhihengliuella, Youngiibacter, Weissella, Virgibacillus, Vibrio, Veillonella, Vagococcus, Undibacterium, Turicibacter, Trichococcus, Thiomonas, Tepidiphilus, Tepidimonas, Syntrophococcus, Symbiobacterium, Sutterella, Sunxiuqinia, Succinivibrio, Streptococcus, Sporomusa, Sphingobacterium, Solobacterium, Simplicispira, Shewanella, Schwartzia, Saccharicrinis, Ruminococcus, Rugamonas, Rothia, Rikenella, Rheinheimera, Ramlibacter, Psychrilyobacter, Proteiniphilum, Propionigenium, Prolixibacter, Porphyromonas, Phocaeicola, Phascolarctobacterium, Peptostreptococcus, Pelosinus, Parvimonas, Parasutterella, Paraeggerthella, Paenibacillus, Oxalicibacterium, Oscillibacter, Olsenella, Neisseria, Mogibacterium, Methylovorus, Leuconostoc, Leptotrichia, Leminorella, Lactobacillus, Lachnospira, Howardella, Holdemania, Granulicatella, Gibbsiella, Gemmiger, Gemella, Filifactor, Faecalicoccus, Eubacterium, Dysgonomonas, Desulfovibrio, Desulfotomaculum, Desulfitibacter, Defluviitalea, Corynebacterium, Coprobacillus, Clostridium, Christensenella, Cellulosilyticum, Catenisphaera, Campylobacter, Caloramator, Buttiauxella, Bulleidia, Bacteroides, Atopobium, Anoxybacillus, Anaerovibrio, Anaerosporobacter, Anaerobiospirillum, Anaerobacter, Allisonella, Actinomyces, Acetatifactor

2.3 QNetDiff Score and Other Scores for Representative Bacteria

The proposed method was applied to the data obtained from stool samples of 576 colorectal cancer patients, and finally 26 genera remained as the core bacteria and their related bacteria. Here, in Supplementary Table 5, we present the QNetDiff scores and other scores for each of these 26 genera.

Supplementary Table 5. QNetDiff score, NetShift score, *p*-value and average abundance of all 26 bacteria constituting the bacterial correlation network consisted of core bacteria and their related bacteria.

Bacteria	QNetDiff score	NetShift score	*p*-value	degree in Healthy	degree in Stage_0	ave. abund. in Healthy	ave. abund. in Stage_0
Actinomyces	7.041117	2.066667	0.000219	2.867917	9.909034	0.000383	0.000812
Granulicatella	7.010286	2.000000	0.029593	3.438871	10.415549	0.000205	0.000416
Gemella	6.289972	2.148459	0.001938	1.949498	8.239470	0.000250	0.000456
Parvimonas	3.692600	2.380952	0.017119	0.000000	3.692600	0.000075	0.000131
Atopobium	3.394089	2.083333	0.000045	0.448282	3.842371	0.000059	0.000160
Enterococcus	3.361818	1.500000	0.306602	2.504242	5.756945	0.001044	0.000596
Rothia	3.166174	1.285714	0.185611	2.807000	5.863229	0.000191	0.000201
Clostridium	2.649021	1.488095	0.118788	1.464141	4.091439	0.046663	0.051222
Neisseria	2.544827	2.285714	0.253039	0.000000	2.544827	0.000033	0.000132
Providencia	2.471823	1.238095	0.134880	2.288419	4.747660	0.000092	0.000121
Solobacterium	2.405675	2.238095	0.000012	0.000000	2.405675	0.000092	0.000154
Vibrio	2.067407	1.079365	0.328844	2.533648	4.485316	0.000369	0.000670
Shigella	1.930256	1.079365	0.438495	2.257948	4.188204	0.005100	0.004662
Roseburia	1.916198	1.790476	0.975191	0.520166	2.436365	0.031584	0.023574
Filifactor	1.901216	1.523810	0.000755	1.086411	2.987627	0.000145	0.000289
Veillonella	1.814904	0.742857	0.106837	3.277765	5.020985	0.006833	0.005116
Peptostreptococcus	1.572020	1.142857	0.025170	1.298182	2.870202	0.000088	0.000174
Fusobacterium	1.470803	1.342857	0.000029	1.128434	2.577513	0.011000	0.021916
Haemophilus	1.433671	0.688312	0.137658	3.692837	5.029864	0.004757	0.005542
Corynebacterium	1.343562	2.142857	0.012035	0.000000	1.343562	0.000031	0.000042
Streptococcus	1.049759	0.229437	0.246022	5.072468	5.964485	0.019159	0.015476
Psychrilyobacter	0.981180	2.095238	0.018245	0.000000	0.981180	0.000007	0.000057
Mogibacterium	0.932325	2.095238	0.000416	0.000000	0.932325	0.000107	0.000165
Leptotrichia	0.838290	2.095238	0.003488	0.000000	0.838290	0.000021	0.000041
Ruminococcus	0.646861	0.714286	0.656060	0.930197	1.577058	0.035778	0.037941
Bulleidia	0.476066	2.047619	0.000184	0.000000	0.476066	0.000024	0.000037
